# Noninvasive Continuous Glucose Monitoring Using a Multisensor-Based Glucometer and Time Series Analysis

**DOI:** 10.1038/s41598-017-13018-7

**Published:** 2017-10-04

**Authors:** Zhanxiao Geng, Fei Tang, Yadong Ding, Shuzhe Li, Xiaohao Wang

**Affiliations:** 1 State Key Laboratory of Precision Measurement Technology and Instruments, Department of Precision Instrument, Tsinghua University, Beijing, 100084 China; 2 Division of Advanced Manufacturing, Graduate School at Shenzhen, Tsinghua University, Shenzhen, 518055 China

## Abstract

Daily continuous glucose monitoring is very helpful in the control of glucose levels for people with diabetes and impaired glucose tolerance. In this study, a multisensor-based, noninvasive continuous glucometer was developed, which can continuously estimate glucose levels via monitoring of physiological parameter changes such as impedance spectroscopy at low and high frequency, optical properties, temperature and humidity. Thirty-three experiments were conducted for six healthy volunteers and three volunteers with diabetes. Results showed that the average correlation coefficient between the estimated glucose profiles and reference glucose profiles reached 0.8314, with a normalized root mean squared error (NRMSE) of 14.6064. The peak time of postprandial glucose was extracted from the glucose profile, and its estimated value had a correlation coefficient of 0.9449 with the reference value, wherein the root mean square error (RMSE) was 6.8958 min. Using Clarke error grid (CEG) analysis, 100% of the estimated glucose values fell in the clinically acceptable zones A and B, and 92.86% fell in zone A. The application of a multisensor-based, noninvasive continuous glucometer and time series analysis can endure the time delay between human physiological parameters and glucose level changes, so as to potentially accomplish noninvasive daily continuous glucose monitoring.

## Introduction

As a global chronic disease, diabetes is associated with high morbidity^1^ and cannot be fully cured with current technology^2^. Within the years before onset, diabetes manifests as impaired glucose tolerance (IGT). People with IGT comprise an even larger group than those with diabetes and suffer a significantly increased risk of cardiovascular disease (CVD) compared with people with normal glucose^3^. Testing the postprandial glucose profile is an important method of evaluating glucose tolerance^4^. Researchers have shown that a delayed peak time of postprandial glucose indicates degraded cellular function and worse glucose tolerance^5,6^. Continuous glucose monitoring can promote the timely detection of IGT and thus, the adjustment of lifestyle to prevent or delay the progression to diabetes^4,5,7–10^. For those with diagnosed diabetes, continuous glucose monitoring can play a role in monitoring the therapeutic effect during the treatment process, so as to regulate the therapeutic strategy in a timely manner. Therefore, it will be of great use for IGT and diabetes if daily continuous glucose self-monitoring can be realized.

Currently there are two main methods to continuously monitor glucose. One is to perform finger stick glucose monitoring at specified time intervals (for example, once every 30 min). This method will cause physical discomfort and infection risk, which is not suitable for daily, frequent self-monitoring. The other method is to use a dynamic glucometer such as DexcomG4, Medtronic CGMS GOLD etc. The probe of the dynamic glucometer must be inserted into the human body at the hospital, which will also cause physical discomfort and infection risk. Moreover, the probe is for one-time use only, with a service life of only about 3 days; therefore, this method is subject to high cost and is not suitable for long-term, daily continuous glucose monitoring^11–15^.

Noninvasive glucose monitoring can accomplish painless, risk-free, low-cost and frequent testing of glucose, which is an ideal method for self-monitoring of glucose^16^. Noninvasive glucose test methods mainly include the reverse iontophoresis method^17^, optics-related method^18–23^, metabolic heat conformation method^24,25^, bio-impedance spectroscopy method^26^ etc. Due to the complexity of human body composition and physiological processes, there has been no single noninvasive method to date that can achieve sound clinical test results alone; therefore, researchers have conducted their studies on the integration of multiple methods, which has become a major trend for research on noninvasive glucose monitoring. Caduff *et al*. conducted continuous glucose detection by integration of bio-impedance spectroscopy and photoelectric sensors^27–30^. Glucotrack of Integrity Applications from Israel integrated three methods including the heat conduction method, the ultrasonic method and impedance spectroscopy, and each method estimated glucose level independently. A weighted average of the three estimated values was then calculated to obtain the final estimation value^31^. Kiseok Song *et al*. estimated glucose using impedance spectroscopy combined with the infrared spectroscopy method^32^. Jintao Xue *et al*. tested glucose in artificial plasma through a combination of infrared spectroscopy and Raman spectroscopy^33^. For the above noninvasive glucose monitoring methods, the main model is to obtain the glucose value according to physiological parameters at the time of testing^25,27–31^. Researchers have evaluated the time lag between blood and interstitial fluid glucose^34–36^. Also, there is a time delay between changes in glucose and changes in physiological parameters, and the time delay may vary from one physiological parameter to another. In fact, the physiological parameters at the time of testing should be related to the glucose concentration over a period of time. As the aim of most noninvasive methods is to obtain glucose values by analysing the influence of glucose on physiological parameters, it is not correct to obtain the glucose value by simply analysing the physiological parameters at the time of testing.

In this paper, a multisensor-based, noninvasive continuous glucometer integrated with impedance spectroscopy at low and high frequency, optical properties, temperature and humidity was developed, which can estimate glucose variation by continuously obtaining time series data from multiple sensors. By screening features according to the similarity between each feature and the reference glucose profile and establishing a model using time series analysis, this proposed glucometer overcame the problem of the time delay between physiological parameter changes and glucose level changes. By conducting experiments on diabetic and nondiabetic, we showed that such a glucometer could potentially realize noninvasive, daily, continuous glucose monitoring.

## Results

### Continuous glucose profiles

**Figure 1:**
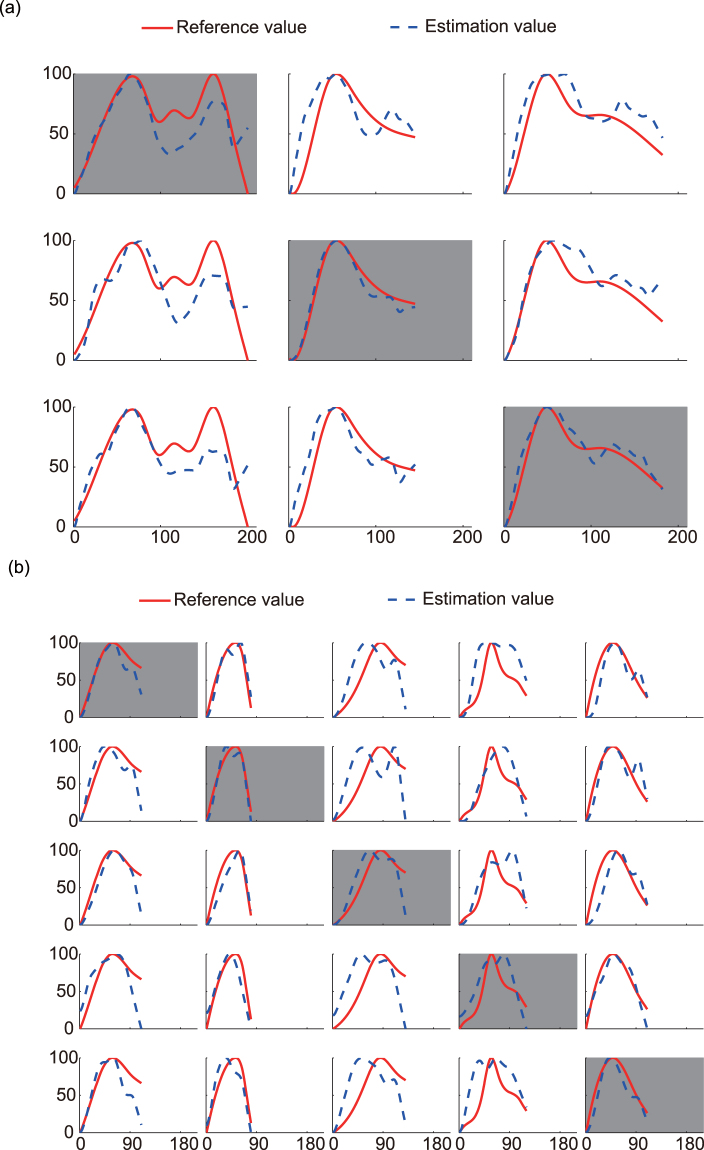
Experiment results of (**a**) healthy volunteer 1 and (**b**) diabetes volunteer 1. N-Value is the normalized glucose value. For each row, the grey background figures are modelling results, and the white background figures are estimated glucose results. The estimation value is the noninvasive measured glucose profile, the reference value is the reference glucose profile.

To evaluate the ability of noninvasive continuous glucose monitoring, the continuous glucose profiles were obtained by the multisensor-based glucometer. In total, three volunteers with diabetes, each of them accepting to participate in five experiments, and six healthy volunteers, each of them accepting to participate in three lunch experiments, were recruited. The reference glucose profiles were measured either by dynamic glucometer (for volunteers with diabetes) or finger stick (for healthy volunteers). For each volunteer, one group of experimental data was selected for modelling, while the remaining groups of data were used for estimation. There were three groups of modelling and six groups of estimations for each healthy volunteer, as shown in Fig. 1(a), in which the grey background figures are modelling results, and the white background figures are estimated glucose results. For each volunteer with diabetes, there are 5 groups of modelling and 20 groups of estimations, as shown in Fig. 1(b).

For all estimated glucose profiles, we calculated the correlation coefficient, NRMSE (normalized root mean squared error) and MAPE (mean absolute percent error) of each estimation result. NRMSE and MAPE were as follows:

1 $$NRMSE=\sqrt{\frac{\sum {(Glu(t)-G(t))}^{2}}{N}}$$

2 $$MAPE=\frac{1}{N}\sum |\frac{Glu(t)-G(t)}{Glu(t)}|$$

where *Glu*(t) was the real glucose value, *G*(t) was the estimation glucose value and *N* is the number of test points.

The average results for each volunteer are shown in Table 1.

**Table 1:** Experiment results.

Volunteer ID	Correlation	RMSE	MAPE
Healthy volunteer 1	0.8619	11.4315	0.0892
Healthy volunteer 2	0.9039	10.6838	0.0638
Healthy volunteer 3	0.9330	11.3526	0.1127
Healthy volunteer 4	0.7796	15.0616	0.0936
Healthy volunteer 5	0.8208	14.7912	0.0835
Healthy volunteer 6	0.7938	14.7370	0.1259
Diabetes volunteer 1	0.7996	16.9059	0.1128
Diabetes volunteer 2	0.7908	19.5886	0.1121
Diabetes volunteer 3	0.7996	16.9059	0.1128

From the table, we can see that the highest correlation coefficient reached 0.9330, while the lowest was 0.7796. The average value of all correlation coefficients was 0.8314, the average NRMSE was 14.6064 and the average MAPE was 0.1128. The NRMSE ranged from 10 to 20, MAPE was approximately 0.1. The NRMSE and MAPE values of diabetes volunteers were slightly larger than those of healthy volunteers, which indicated that the relative error between estimation glucose value and reference glucose value is approximately 10%, and the model showed sound adaptation to both volunteers with diabetes and healthy volunteers.

### Postprandial glucose peak time

**Figure 2:**
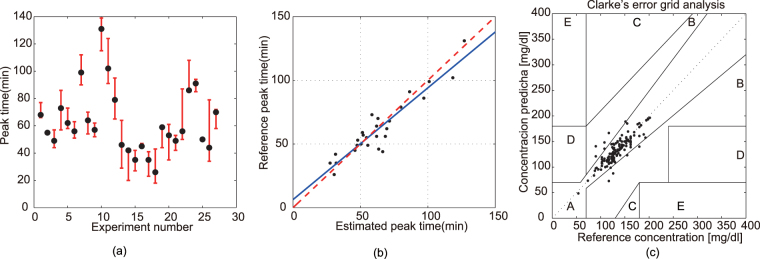
Peak time and Clarke error gird result. (**a**) The comparison between RPT and Max-EPT and Min-EPT, wherein the block dot was RPT. (**b**) The comparison between RPT and average value of estimation PT. (**c**) Clarke’s error grid result of 140 finger stick monitoring points. 92.86% of the estimated glucose values fell in zone A and 100% in the clinically acceptable zones A and B.

Following consumption of the standard lunch, the peak time (PT) of postprandial glucose is closely correlated to glucose tolerance^5,6^. There were three standard catering experiments for both healthy volunteers and volunteers with diabetes. The estimation PTs in all 27 standard catering experiments were analysed. For each experiment, the remaining experiments on the same volunteer were used for modelling, and then the Max-EPT (max estimated peak time) and Min-EPT (min estimated peak time) were selected for comparison with the RPT (reference peak time), as shown in Fig. 2(a), showing that the estimated PT fluctuates around the real PT. Figure 2(b) showed the comparison between RPT and the average estimated value of PT, wherein the correlation coefficient was 0.9449, the root mean square error was 6.8958 min, the max lag was 18.5 min and the max advance was 14.5 min. The estimated PT was highly correlated with the reference PT.

### Glucose value at certain points

In this experiment, 33 tests were conducted on nine volunteers, collecting the noninvasive glucose value at a total of 140 finger stick monitoring points. In each glucose estimation profile, two reference glucose points were selected for calibration, and thus a glucose estimation profile that characterized the real glucose value was obtained. According to this profile, we estimated the glucose values at the remaining test points and compared them with the reference values, thereby obtaining the Clarke error grid as shown in Fig. 2(c).

In the Clarke error grid, 92.86% of the estimated glucose values fell in zone A and 100% in the clinically acceptable zones A and B, demonstrating the acceptability of the non-invasive blood glucometer.

## Discussion

Data from 27 standard catering experiments were collected for further analysis, in which correlation coefficient and mean absolute deviation (MAD) were taken as the criteria.

**Figure 3:**
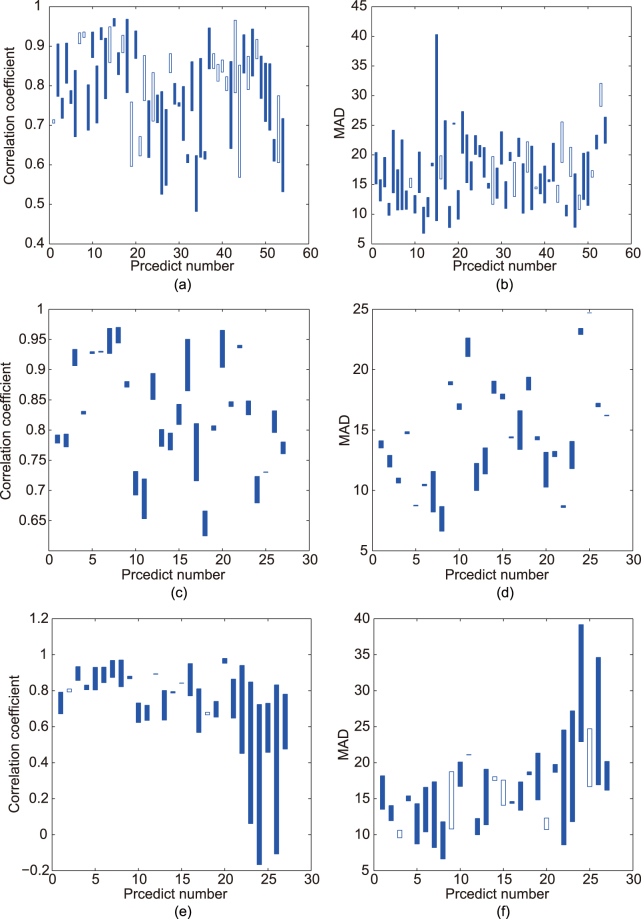
Comparison of different models. (**a**), (**b**) Comparison of the single-feature model and multi-feature fusion. (**c**), (**d**) Comparison of the single-model and multi-model. (**e**), (**f**) Comparison of the self-model and others-model. The left side showed the candlestick charts of the correlation coefficient. When the correlation coefficient of multi-feature fusion, the multi-model and the self-model was higher than that of the single-feature model, single-model and others-model, respectively, the candlestick was solid. The right side showed the candlestick charts of mean absolute deviation. When the mean absolute deviation of multi-feature fusion, the multi-model and the self-model was lower than that of the single-feature model, single-model and others-model, respectively, the candlestick was solid.

To compare the estimation results of the single-feature model and multi-feature fusion, the results of each estimation were investigated separately. We compared the estimation result of multi-feature fusion and the best estimation result of single-feature models. For each volunteer, each group of experimental data was used for modelling, while the remaining groups of data were used for estimation. Therefore, there were three groups of modelling and six groups of estimations for each volunteer. There were 54 estimations in total for nine volunteers. When using single-feature model, the optimal feature obtained from different experiments differed. As shown in Fig. 3(a) and (b), the results of multi-feature fusion were superior to the best results of the single-feature model in most cases. Multi-feature fusion can better use the features, realize better model results, and achieve more stable estimation results as compared with the single-feature model.

In this research, the model established based on the data of one experiment was called a single-model. By integrating two or more single models, one obtained a multi-model. For each volunteer, the estimation curve ${G}_{k}(t)$, *k* = *1*, …, *n*, was obtained using models based on the remaining experiments conducted on the same individual. The correlation coefficient $co{r}_{k}$ and mean absolute deviation $ma{d}_{k}$ between ${G}_{k}(t)$ and the reference glucose profile $Glu(t)$ were calculated.

For the single-model, the average values of $co{r}_{k}$ and $ma{d}_{k}$ were calculated, thus obtaining the final correlation coefficient $co{r}_{s}$ and average mean absolute deviation $ma{d}_{s}$, as shown in equation (3) and (4). For the multi-model, the mean value of ${G}_{k}(t)$ was calculated, thus obtaining the final estimation glucose profile ${G}_{m}(t)$, as shown in equation (5). The correlation coefficient $co{r}_{m}$ and mean absolute deviation $ma{d}_{m}$ between ${G}_{m}(t)$ and reference glucose profile $Glu(t)$ were calculated.

3 $$co{r}_{s}=\frac{{\sum }_{k=1}^{n}\,co{r}_{k}}{n}$$

4 $$ma{d}_{s}=\frac{{\sum }_{k=1}^{n}\,ma{d}_{k}}{n}$$

5 $${G}_{m}(t)=\frac{{\sum }_{k=1}^{n}\,{G}_{k}(t)}{n}$$

In this research, each volunteer participated in three standard catering experiments, thus *n* = 2. Figure 3(c) and (d) showed the comparison of the correlation coefficient and mean absolute deviation between the single- and multi-model, from which we can see that the results of the multi-model are better than the average value of the single model.

The model established based on individual’s experimental data is called the self-model, whereas the model established based on others’ experimental data is called the others-model. To compare the self-model result and the others-model result, each experiment was investigated individually. For the three experiments conducted on one volunteer, to estimate the results of a given experiment, we used the multi-model results as self-model estimation curves; regarding the estimation curves of the others-model, we used the data from the 24 experiments conducted on the other eight volunteers to build models, and then calculated the mean values of these models’ estimation curves as the others-model estimation curves.

As shown in Fig. 3(e) and (f), under the condition that the others-model adopts larger sample database, the self-model can achieve better result than the others-model in most experiments. Particular for diabetes volunteers (experiments 19–27), the results of the others-model are even worse.

Analysis results showed that the model based on multiple features (multi-feature fusion) is more stable than that based on a single feature (single-feature model); moreover, there was actually no fixed optimal single feature. In most situations, the multi-feature fusion was better than the single-feature model. Although an estimation model can be established based on data from one experiment (single-model) by using the method proposed in this paper, the model based on data from multiple experiments (multi-model) was better. The model based on individual’s data (self-model) was superior to that based on others’ experiment data (others-model), especially for people with diabetes.

Figure 1(a) and (b) show significant discrepancies in a number of the measurement profiles between reference and estimated values. There are some characteristics in these discrepancies. First, the discrepancies vary from person to person. The result is better for certain experiment. Taking Fig. 1(a) as an example, the second and third experiment (second and third column) are better than the first experiment (first column). Second, the discrepancies vary between healthy volunteers and the diabetes. Comparing Fig. 1(a), Figs S1–S5 with Fig. 1(b), Figs S6–S7, we can find that the results are better for the healthy volunteers.

We analyzed the original data thoroughly, the relations between physical parameters and glucose levels are slightly changing during the test. It may be caused by the non-glucose-effect physical parameter changes. That effect makes the results fluctuant. Also, the diabetes volunteers were inpatients of the endocrinology. For safety purpose, we did not change the diabetes’ normal treatment program, which may have effect on the diabetes’ results.

**Figure 4:**
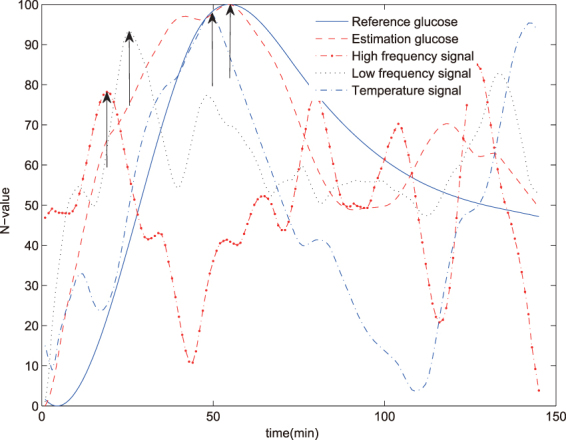
The time delay between glucose and physical parameters of Healthy volunteer 1. The black arrows showed the peak time of glucose and physiological parameters. The estimation glucose profile peak time was close to the peak time of reference glucose profile, and they were indicated in one arrow.

To evaluate the time delay between human physiological parameters and glucose level changes, the original sensor signals, the reference glucose profile and the estimation glucose profile were analysed. The data of Healthy volunteer 1 was used. High frequency signal, low frequency signal and temperature signal were select as examples to indicate the time delay. The estimation glucose profile peak time was close to the peak time of reference glucose profile, and they were indicated in one arrow. It clearly showed the different time delay of different parameters in Fig. 4. The result confirmed the truth that the noninvasive continuous glucometer and time series analysis can endure the time delay between human physiological parameters and glucose level changes.

## Methods

### Noninvasive continuous glucometer

**Figure 5:**
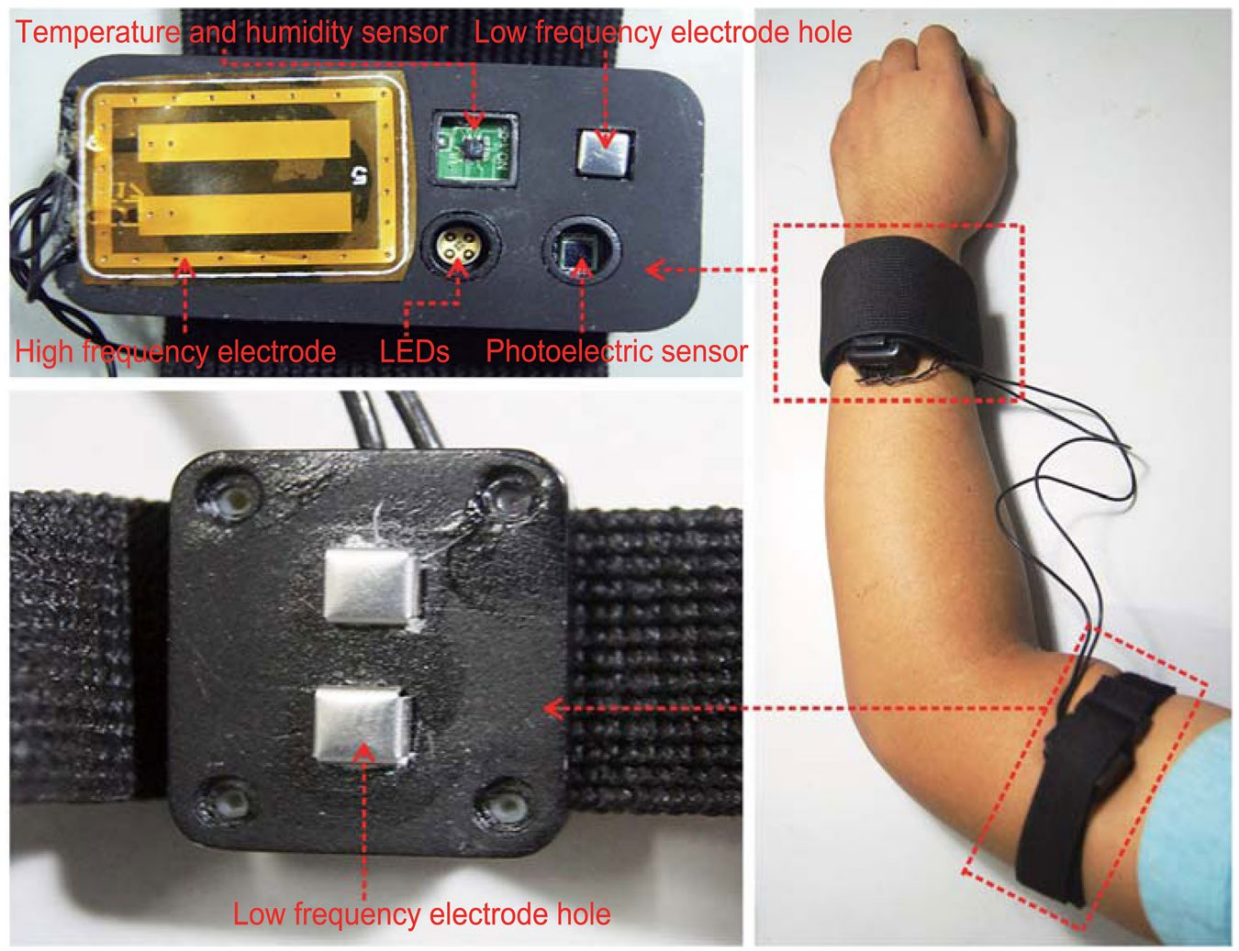
The multisensor-based noninvasive continuous glucometer. The left part was the multisensor probes (the upper one is the larger probe, and the bottom one is the smaller probe), the right part was a sketch map showing how to wear the probes.

Previous work shows that the optical^26,27,37^ and electrical^13,18,20,21^ characteristics of skin, temperature and humidity^24,25^ are all related to changes in glucose. In this paper, two multisensor probes (one larger and the other small) were designed, which can be put on the wrist and upper arm as shown in Fig. 5. The larger one was equipped with light-emitting diodes (LEDs), a photoelectric sensor, temperature sensor, humidity sensor, high frequency flexible electrode and one pole of a low-frequency electrode. The smaller one was equipped with the other pole of the low-frequency electrode, which can be used with the larger probe to detect the low-frequency impedance of the arm.

The wavelengths of the LEDs are 660 nm, 730 nm, 800 nm and 940 nm. The size of the high- frequency electrode is 30 mm × 20 mm, the distance between the two poles is 4 mm, the frequency range is 10–60 MHz and the matching inductance is 220 nH. The low-frequency electrode is made of stainless steel of size 5 mm × 5 mm, the distance between the two poles during operation is approximately 25 cm and the frequency range is 1–150 KHz.

### Modelling

#### Algorithm framework

**Figure 6:**
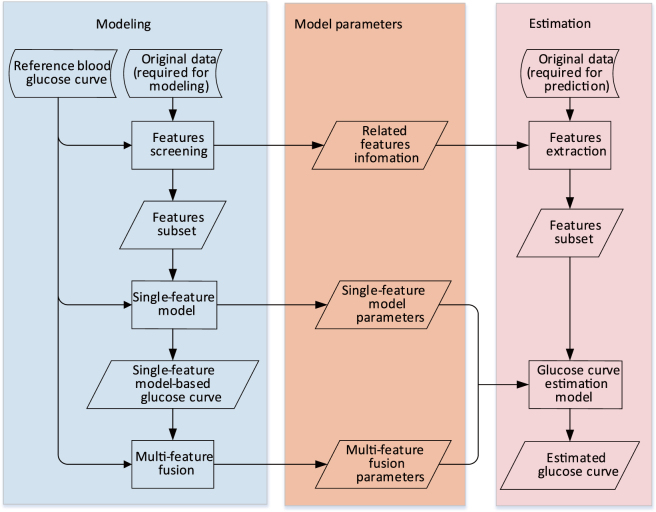
The algorithm framework. The left part showed how to obtain model parameters through modelling, and the right part showed how to obtain estimated glucose profile using model parameters.

Figure 6 showed the algorithm framework, which can be divided into a modelling part and an estimation part. The modelling part can be divided into four steps. First, the reference glucose profile and original data required for modelling were input. Second, all candidate features were calculated from original data, features were screened according to the similarity between the feature and reference glucose profile, added in the related features subset and recorded in related features information. Third, the single-feature model was constructed. The model was established based on each related feature using time series analysis, and then single-feature model parameters and single-feature model-based glucose profiles were obtained. Finally, multi-feature fusion was carried out. Single-feature model-based glucose profiles were integrated using the weighted average method, and then multi-feature fusion parameters were obtained. Single-feature model parameters and multi-feature fusion parameters constituted the glucose profile estimation model.

When estimating the glucose profile, the original data required for estimation were input into the system, the related features were extracted according to related features information obtained during the modelling process, and then the related features subset was input into the estimation model to obtain the estimated glucose profile. After the estimated glucose profile was obtained, the peak time of postprandial glucose can be obtained from it.

#### Features screening

Extracting glucose-related features (hereinafter referred to as related features) from all candidate features is a very important step in noninvasive glucose research. In this paper, a cross-correlation function was used to realize features screening.

A CCF (cross-correlation function) is a measure of the similarity of two series as a function of the displacement of one relative to the other. $y(n)$ is the time series of reference glucose, $x(n)$ is the time series of a certain feature, $\tau$ is the displacement. The CCF can be described by equation (6).

6 $$R(\tau )=\sum _{m=0}^{N-1}\frac{x(m)y(m+\tau )}{N}$$

where $R(\tau )$ is the CCF value. Normally, CCF is used to determine the time delay between two signals according to the maximum value, ${R}_{\max }$. In features screening, when the maximum CCF value exceed the threshold value, the two signals are regarded as similar and the feature is selected as a related feature.

In order to eliminate the influence of random disturbance, the original time series was first subjected to wavelet filtering before calculating the CCF.

#### Single-feature model and multi-feature fusion

In one experiment, an estimation glucose profile can be obtained based on each related feature, which is called a single-feature model. By performing a weighted average algorithm for all estimation glucose profiles based on each related feature, one can obtain a multi-feature-based estimation glucose profile, which is called multi-feature fusion.

Since the feature at the time of measurement is probably related to the glucose level of the preceding period, time series analysis was used to establish a single-feature model. A moving average (MA) model was used to express the relation between the related feature and the reference glucose value, as shown in equation (7):

7 $$Glu(t)=\sum _{n=0}^{m-1}{x}_{i}(t-n)\times {b}_{in}+{\varepsilon }_{i}(t)$$

where *Glu* (t) is the reference glucose value at *t*, *i* is the serial number of the related feature, *x*_*i*_(*t* − *n*) is the value of related feature at *t* − *n*, *b*_in_ is the model coefficient, *m* is the model order and ε_*i*_(t) is residual error.

The result of the MA model is ${g}_{i}(t)$. The time delay *T*_*i*_ between ${g}_{i}(t)$ and $Glu(t)$ is calculated by CCF, and then *T*_*i*_ is eliminated to obtain the final single-feature model-based glucose profile ${G}_{i}(t)$, as shown in equation (8):

8 $${G}_{i}(t)={g}_{i}(t-{T}_{i})$$

In multi-feature fusion, a weighted average algorithm was performed to fuse all single-feature model-based glucose profiles ${G}_{i}(t)$, and finally obtain $G(t)$ as shown in equation (9):

9 $$G(t)={G}_{i}(t)\times {K}_{i}$$

where $G(t)$ is the estimated glucose profile based on multi-feature fusion, and ${K}_{i}$ is the weight of the single-feature model-based glucose profile calculated using the *ith* related feature.

In the modelling process, the single-feature model parameters can be obtained as *T*_*i*_, *b*_*in*_ and *m*, and the multi-feature fusion parameters can be obtained as *K*_*i*_. During glucose estimation, features are extracted from related features information and added into the features subset. The estimated glucose profile can be obtained using single-feature model parameters and multi-feature fusion parameters.

### Experimental design

To verify the effectiveness of the algorithm, healthy volunteers and volunteers with diabetes were recruited for multiple experiments. This experiment was approved by the ethics committee of the Peking University First Hospital, and all methods were performed in accordance with the relevant guidelines and regulations. In total, three volunteers with diabetes, each of them accepting to participate in five experiments, and six healthy volunteers, each of them accepting to participate in three lunch experiments, were recruited. Volunteers with diabetes were inpatients wearing a dynamic glucometer (Medtronic, MiniMed Paradigm 722) for a continuous 72 h of monitoring. Informed consent was obtained from all participants. Each of them was subjected to three lunch experiments and two supper experiments, wherein the lunch was standardized and included 90 g of standard tortilla, while the supper was without specific requirements. Healthy volunteers were subjected to only the standard lunch experiment once every week without wearing the dynamic glucometer.

At 10 min before the meal, volunteers wore the developed noninvasive continuous glucometer and underwent finger stick glucose monitoring (Roche glucometer, ACCU-CHEK® Performa) once for reference fasting glucose. The timer was then started; meal time should be controlled to within 10–15 min. Finger stick glucose monitoring was performed once every 30 min. During the experiment, volunteers were kept indoors and asked not to perform strenuous exercises. Volunteers with diabetes were not prevented from using hypoglycaemic drugs or insulin injection. For healthy volunteers, the reference glucose profiles were obtained by interpolating through the finger stick point. For volunteers with diabetes, the reference glucose profiles were obtained by the dynamic glucometer.

### Data availability

The datasets generated during and/or analysed during the current study are available from the corresponding author on reasonable request.

## Electronic supplementary material

Supplementary material

## References

[1] Guariguata, L. *et al*. Global estimates of diabetes prevalence for 2013 and projections for 2035 for the IDF Diabetes Atlas. *Diabetes Research and Clinical Practice*, 137–149 (2013). 10.1016/j.diabres.2013.11.002 24630390

[2] Zhang, M., Xu, W. & Deng, Y. A New Strategy for Early Diagnosis of Type 2 Diabetes Mellitus by standard-free, label-free LC-MS/MS quantification of glycated peptides. *Diabetes*, DB_130347 (2013). 10.2337/db13-0347 PMC3806625 23894188

[3] Tominaga M (1999). Impaired glucose tolerance is a risk factor for cardiovascular disease, but not impaired fasting glucose. The Funagata Diabetes Study. Diabetes care.

[4] Cavalot F (2006). *Postprandial blood glucose is a stronger predictor of* cardiovascular events than fasting blood glucose in type 2 diabetes mellitus, particularly in women: lessons from the San Luigi Gonzaga Diabetes Study. The Journal of Clinical Endocrinology & Metabolism.

[5] Daenen S (2010). *Peak-time determination* of post-meal glucose excursions in insulin-treated diabetic patients. Diabetes & metabolism.

[6] Kramer CK (2015). Delayed timing of post-challenge peak blood glucose predicts declining beta cell function and worsening glucose tolerance over time: insight from the first year postpartum. Diabetologia.

[7] Cartee GD, Funai K (2009). Exercise and insulin: Convergence or divergence at AS160 and TBC1D1?. Exercise and sport sciences reviews.

[8] Fang Z (2014). Shenzhu Tiaopi granule combined with lifestyle intervention therapy for impaired glucose tolerance: A randomized controlled trial. Complementary therapies in medicine.

[9] Fontana L, Klein S, Holloszy JO (2010). Effects of long-term calorie restriction and endurance exercise on glucose tolerance, insulin action, and adipokine production. Age.

[10] Tuomilehto J (2001). Prevention of type 2 diabetes mellitus by changes in lifestyle among subjects with impaired glucose tolerance. New England Journal of Medicine.

[11] Battelino T, Bolinder J (2008). Clinical use of real-time continuous glucose monitoring. Current Diabetes Reviews.

[12] Garg S (2006). Improvement in glycemic excursions with a transcutaneous, real-time continuous glucose sensor a randomized controlled trial. Diabetes care.

[13] Sparacino G, Facchinetti A, Cobelli C (2010). “Smart” continuous glucose monitoring sensors: on-line signal processing issues. Sensors.

[14] Sparacino, G., Facchinetti, A., Zecchin, C. & Cobelli, C. In *XIII Mediterranean Conference on Medical and Biological Engineering and Computing 2013*. 1543–1546 (Springer).

[15] Vashist SK (2013). Continuous glucose monitoring systems: A review. Diagnostics.

[16] Vashist SK (2012). Non-invasive glucose monitoring technology in diabetes management: A review. Analytica chimica acta.

[17] Tamada JA (1999). Noninvasive glucose monitoring - Comprehensive clinical results. Jama-J Am Med Assoc.

[18] Alexeeva NV, Arnold MA (2010). Impact of tissue heterogeneity on noninvasive near-infrared glucose measurements in interstitial fluid of rat skin. Journal of diabetes science and technology.

[19] Heinemann L, Schmelzeisen-Redeker G (1998). Non-invasive continuous glucose monitoring in Type I diabetic patients with optical glucose sensors. Diabetologia.

[20] Pleitez MA (2012). *In vivo* noninvasive monitoring of glucose concentration in human epidermis by mid-infrared pulsed photoacoustic spectroscopy. Analytical chemistry.

[21] Sarangi, S., Pai, P. P., Sanki, P. K. & Banerjee, S. In *2014 IEEE 2*7th Inte*rnational Symposium on Computer-Based Medical Systems*. 485–486 (IEEE).

[22] Pandey R (2017). Noninvasive Monitoring of Blood Glucose with Raman Spectroscopy. Acc. Chem. Res.

[23] Chaiken J (2010). Instrument for near infrared emission spectroscopic probing of human fingertips *in vivo*. Review of Scientific Instruments.

[24] Cho OK, Kim YO, Mitsumaki H, Kuwa K (2004). Noninvasive measurement of glucose by metabolic heat conformation method. Clinical Chemistry.

[25] Tang F, Wang X, Wang D, Li J (2008). Non-invasive glucose measurement by use of metabolic heat conformation method. Sensors.

[26] Gelao G, Marani R, Carriero V, Perri AG (2012). Design of a dielectric spectroscopy sensor for continuous and non-invasive blood glucose monitoring. International Journal of Advances in Engineering & Technology.

[27] Caduff A (2015). The Effect of a Global, Subject, and Device-Specific Model on a Noninvasive Glucose Monitoring Multisensor System. Journal of diabetes science and technology.

[28] Caduff A (2006). Non-invasive glucose monitoring in patients with diabetes: A novel system based on impedance spectroscopy. Biosensors and Bioelectronics.

[29] Caduff A (2009). Non-invasive glucose monitoring in patients with Type 1 diabetes: A Multisensor system combining sensors for dielectric and optical characterisation of skin. Biosensors and Bioelectronics.

[30] Caduff A (2011). Characteristics of a multisensor system for non invasive glucose monitoring with external validation and prospective evaluation. Biosensors and Bioelectronics.

[31] Harman-Boehm I, Gal A, Raykhman AM, Naidis E, Mayzel Y (2010). Noninvasive glucose monitoring: increasing accuracy by combination of multi-technology and multi-sensors. Journal of diabetes science and technology.

[32] Song, K., Ha, U., Park, S. & Yoo, H.-J. In *VLSI Circuits Digest of Technical Papers*, *2014**Symposium on*. 1–2 (IEEE).

[33] Xue JT (2014). Noninvasive Measurement of Glucose in Artificial Plasma with Near-Infrared and Raman Spectroscopy. Applied Spectroscopy.

[34] Barman I, Kong C-R, Singh GP, Dasari RR, Feld MS (2010). Accurate spectroscopic calibration for noninvasive glucose monitoring by modeling the physiological glucose dynamics. Analytical chemistry.

[35] Schmidtke DW, Freeland AC, Heller A, Bonnecaze RT (1998). Measurement and modeling of the transient difference between blood and subcutaneous glucose concentrations in the rat after injection of insulin. Proceedings of the National Academy of Sciences.

[36] Spegazzini, N. *et al*. Spectroscopic approach for dynamic bioanalyte tracking with minimal concentration information. *Scientific reports* 4 (2014). 10.1038/srep07013 PMC4894421 25388455

[37] Song K, Ha U, Park S, Bae J, Yoo HJ (2015). An Impedance and Multi-Wavelength Near-Infrared Spectroscopy IC for Non-Invasive Blood Glucose Estimation. Ieee J Solid-St Circ.

