# Supplementary material for: Noninvasive Continuous Glucose Monitoring Using a Multisensor-Based Glucometer and Time Series Analysis
Source: Sci Rep. 2017 Oct 4;7:12650. doi: 10.1038/s41598-017-13018-7 (PMC5627266; doi:10.1038/s41598-017-13018-7)
Supplement: Supplementary file 1 — Supplementary material [file 41598_2017_13018_MOESM1_ESM.doc]

**Noninvasive Contunious Glucose Monitoring Using a Multisensor-Based Glucometer and Time Series Analysis**

Zhanxiao GENG ^a^, Fei TANG ^a^, *, Yadong DING ^a^, Shuzhe LI ^a^ and Xiaohao WANG ^a, b^

The following figures are the volunteer’s experimental results other than the ones showed in the manuscript. The red solid lines are reference glucose measured by Roche glucometer, ACCU-CHEK® Performa. The blue dashed lines are noninvasively estimated glucose. N-Value is the normalized glucose value. For each row, the grey background figures are modelling results, and the white background figures are estimated glucose results.

Figure S1 Healthy volunteer 2

Figure S2 Healthy volunteer 3

Figure S3 Healthy volunteer 4

Figure S4 Healthy volunteer 5

Figure S5 Healthy volunteer 6

Figure S6 Diabetes volunteer 2

Figure S7 Diabetes volunteer 3

The following figure were the Bland Altman plots for healthy volunteer 1 and diabetes volunteer 1. In Fig S8 and Fig S9, the dotted line represents 1.96 times of the standard deviation.

Figure S8 Bland Altman plots for healthy volunteer 1

Figure S9 Bland Altman plots for diabetes volunteer 1
